# Role of a Genetic Variant on the 15q25.1 Lung Cancer Susceptibility Locus in Smoking-Associated Nasopharyngeal Carcinoma

**DOI:** 10.1371/journal.pone.0109036

**Published:** 2014-10-20

**Authors:** Xuemei Ji, Weidong Zhang, Jiang Gui, Xia Fan, Weiwei Zhang, Yafang Li, Guangyu An, Dakai Zhu, Qiang Hu

**Affiliations:** 1 Department of Radiation Oncology, Beijing Chaoyang Hospital, Capital Medical University, Beijing, China; 2 Department of Community and Family Medicine, Geisel School of Medicine at Dartmouth, Dartmouth College, Hanover, NH, United States of America; 3 Department of Radiation and Medical Oncology, Zhongnan Hospital, Wuhan University, Wuhan, China; 4 Department of Anesthesia, Binzhou Medical University, Binzhou, China; 5 Department of Oncology, Beijing Chaoyang Hospital, Capital Medical University, Beijing, China; IRCCS National Cancer Institute, Italy

## Abstract

**Background:**

The 15q25.1 lung cancer susceptibility locus, containing CHRNA5, could modify lung cancer susceptibility and multiple smoking related phenotypes. However, no studies have investigated the association between CHRNA5 rs3841324, which has been proven to have the highest association with CHRNA5 mRNA expression, and the risk of other smoking-associated cancers, except lung cancer. In the current study we examined the association between rs3841324 and susceptibility to smoking-associated nasopharyngeal carcinoma (NPC).

**Methods:**

In this case-control study we genotyped the CHRNA5 rs3841324 polymorphism with 400 NPC cases and 491 healthy controls who were Han Chinese and frequency-matched by age (±5 years), gender, and alcohol consumption. Univariate and multivariate logistic regression analyses were used to calculate the odds ratio (OR) and 95% confidence intervals (95% CI).

**Results:**

We found that individuals with CHRNA5 rs3841324 combined variant genotypes (ins/del+del/del) had a >1.5-fold elevated risk for NPC than those with the ins/ins genotype (adjusted OR = 1.52; 95% CI, 1.16–2.00), especially among ever smokers (adjusted OR = 2.07; 95% CI, 1.23–3.48). The combined variant genotypes acted jointly with cigarette smoking to contribute to a 4.35-fold increased NPC risk (adjusted OR = 4.35; 95% CI, 2.57–7.38). There was a dose-response relationship between deletion alleles and NPC susceptibility (trend test, P = 0.011).

**Conclusions:**

Our results suggest that genetic variants on the 15q25.1 lung cancer susceptibility locus may influence susceptibility to NPC, particularly for smoking-associated NPC. Such work may be helpful to facilitate an understanding of the etiology of smoking-associated cancers and improve prevention efforts.

## Introduction

Cigarette smoking is a major public health problem, accounting for 5 million deaths annually worldwide [1], and contributing to 31% and 6% of all cancer deaths in men and women worldwide for people between 30 and 69 years of age, respectively [2]. Nicotine, a component of cigarettes, can promote cancer cell proliferation, survival, migration, invasion, etiology, and development [3], Nicotine has been shown to be involved with the pathogenesis of many cancers, including nasopharyngeal carcinoma (NPC) [4]; however, there is no evidence that the carcinogenic mechanisms associated with nicotine and genetic variants influence susceptibility to NPC.

Genome-wide association studies (GWAS) have identified that chromosome 15q25.1, composed of nicotinic acetylcholine receptor genes, including CHRNA5 and CHRNA3, are lung cancer susceptibility regions [5], [6] and play a potential role in multiple smoking-related phenotypes and nicotine dependence [7]. CHRNA5, a member of the ligand-gated ion channels, modulates cell membrane potentials and physiologic processes, including neurotransmission [8] and cancer signaling [9]. Tobacco alkaloid and nicotine can bind and activate nicotinic acetylcholine receptors and thereby stimulate cellular proliferation and tumor invasion, and inhibit apoptosis [9]–[11]. Although it has been reported that nicotine-mediated activation of CHRNA3 and CHRNA5 can influence lung cancer risk directly, it also has been suggested that the 15q25.1 lung cancer susceptibility locus influences lung cancer risk at least in part through an effect on smoking persistence [12] because the variants at 15q25.1 are also associated with smoking behavior and nicotine dependence [13]. Therefore, variants at 15q25.1 might affect other smoking-associated cancers than lung cancer. However, there is only one study that has examined the association between genetic variants at the 15q25.1 lung cancer susceptibility locus (rs8034191 and rs1051730) and the risk of another smoking-associated cancer other than lung cancer (pancreatic cancer) and found no significant association [14]. Therefore, it is unclear whether or not the 15q25.1 lung cancer susceptibility locus is confined to the lung or influences cancer susceptibility related to carcinogenic compound exposure in cigarette smoking.

CHRNA5 rs3841324, a 22 bp insertion (ins)/deletion (del) at position −71 upstream of the transcription start site, has been shown to have the greatest association with CHRNA5 mRNA levels in lung tissue [15], and accounts for approximately 42% of the alteration in CHRNA5 mRNA expression [15]. However, compared to the CHRNA5 rs3841324 ins/ins and ins/del genotypes, the del/del genotype show inconsistent transcription level in different populations. In European descent, the del/del genotype has a 2.9-fold increase in CHRNA5 mRNA levels [15] and significantly reduces the lung cancer risk in female Caucasian ever-smokers [6], while in Han Chinese the del/del genotype is associated with hypoactivity of the promoter and decreased transcription [16]. On the other hand, only the CHRNA5 rs3841324 SNP has been investigated with respect to the possible relationship with lung cancer susceptibility. Therefore, there is an urgent need to investigate whether or not CHRNA5 rs3841324 is involved in susceptibility to smoking-associated cancers in addition to lung cancer.

Our previous large epidemiologic study showed that cigarette smoking is a significant risk factor for NPC [4], which is consistent with previous findings [17], [18]. Thus, to determine whether or not genetic variations in the 15q25.1 lung cancer susceptibility locus are implicated in the carcinogenesis of cigarette smoking-mediated cancer risk other than lung cancer, we evaluated the association between the CHRNA5 rs3841324 polymorphism and NPC risk in general and in subgroups of subjects stratified by age, gender, cigarette smoking, alcohol consumption, and pathology, and explored the joint effect between CHRNA5 rs3841324 and cigarette smoke exposure on NPC risk with 400 patients newly diagnosed with NPC and 491 cancer-free healthy controls. Such work may help facilitate an understanding of the carcinogenic mechanisms by which genetic variants at these regions influence cigarette smoking-associated cancers and the etiology of NPC, and improve prevention efforts.

## Materials and Methods

### Study Subjects

Four hundred newly diagnosed patients, previously untreated with histopathologically-confirmed NPC without age, gender, and cancer stage or histology restrictions, and 491 cancer-free healthy controls who with no history of cancer, were not receiving treatment for any diseases, and were frequency-matched to the cases with respect to age (±5 years), gender, ethnicity, and smoking status (ever or never) were recruited from Zhongnan Hospital of Wuhan University and Beijing Chaoyang Hospital between January 2006 and December 2012. Human participant approval was obtained from the ethical committees of Zhongnan Hospital of Wuhan University and Beijng Chaoyang Hospital of Capital Medical University. To avoid confounding due to ethnic characteristics, we included only Han Chinese in both the case and control groups. All participants signed written informed consent, completed a questionnaire regarding demographic and relevant risk factor information, and donated 3 ml of blood for CHRNA5 genotyping.

### *CHRNA5* Genotyping

We extracted genomic DNA from a leukocyte cell pellet, which was obtained from the buffy coat by centrifugation of 1 ml of whole blood, using the QIAGEN DNA Blood Mini Kit (QIAGEN, Inc., Valencia, CA, USA) according to the manufacturer’s instructions. Agarose gel electrophoresis was used following amplification using a fluorescence polymerase chain reaction (PCR). The fluorescence primer sequences for PCR were as follows: 5′-AGCAGACAGGGTTGGACCAGAG-3′ (forward); and 5′-CGTGAGACAAAACGAGGGCAGAC-3′ (reverse). The PCR amplification was carried out in a final volume of 20 µL containing 10 ng of DNA, 1 µM of each primer, 0.3 mM deoxynucleotide triphosphates (dATP, dCTP, dGTP, and dTTP; each at 0.3 mM), 3.0 mM MgCl_2_, 1x HotStarTaq buffer, and 1 unit of HotStarTaq Polymerase (Qiagen, Inc.). The reaction was started at 95°C for 2 min, followed by 11 cycles of amplification (94°C for 20 sec, 65°C for 40 sec, and 72°C for 90 sec), 24 cycles of amplification (94°C for 20 sec, 59°C for 30 sec, and 72°C for 90 sec), extension at 68°C for 60 min, and 4°C until used. The 15-µL PCR product was added to 5 U SAP enzyme and 2 U exonuclease at 37°C for 60 min, then 75°C for 15 min. The 0.5-µL extension product and 0.5-µL fluorescence PCR product were added to 0.5-µL Lizl 20 SIZE STANDARD and 8.50.5-µL Hi-Di, incubated at 95°C for 5 min, then scanned using an ABI3730XL sequenator. Data from the ABI3730XL sequenator was analyzed using a GeneMapper 4.1 (Applied Biosystems Co., Ltd., USA). The expected size of the PCR product was 267 base pairs (bp) for the rs3841324 ins allele and 245 bp for the del allele.

### Statistical Analysis

Statistical Analysis System software (version 9.1; SAS Institute, Cary, NC, USA) was used for all statistical analyses. Ever-smokers were subjects who had smoked >100 cigarettes in their lifetime, and the remaining smokers were categorized as never-smokers. Ever-drinkers were subjects who drank alcoholic beverages at least once a week for >1 year and the remaining drinkers were categorized as never drinkers. NPC was classified by the World Health Organization (WHO) in 1991 into two categories based on the histologic type, as follows: type I, keratinizing squamous cell carcinoma; and type II, non-keratinizing carcinoma [19]. The differences in the distributions of select demographic variables, smoking status, alcohol consumption, and *CHRNA5* rs3841324 allele and genotype frequencies between the cases and controls were evaluated using a χ^2^ test. The mean values of age were compared for cases and controls using a Student’s *t*-test. We estimated the association between *CHRNA5* genotypes and the risk of NPC by computing the odds ratios (ORs) and 95% confidence intervals (CIs) by univariate and multivariate logistic regression analyses. We further stratified the genotype data by subgroups of age, gender, smoking, alcohol consumption, and pathology of NPC. For logistic regression analysis, the *CHRNA5* genotype was tested with dominant model. We create a dummy variable to represent CHRNA5 coding wild type as 0, heterozygous and rare allele homozygous as 1. In the multivariate logistic regression model, the OR and 95% CI were adjusted by age, gender, smoking, and alcohol consumption. Statistical analyses were performed with Statistical Analysis System softwareand *P* values<0.05 were considered significant.

## Results

For this study, complete *CHRNA5* genotype data, demographic characteristics, and smoking and alcohol consumption status were available for 400 cases with NPC and 491 healthy controls (**Table 1**). No significant differences in age, gender, or alcohol consumption status existed between the NPC patients and healthy controls (P = 0.061 for age, 0.321 for gender, and 0.186 for alcohol consumption). However, the mean age for the patients with NPC (49.7±11.1) was significantly greater than the controls (47.1±9.65; P = 0.0001). Additionally, the distribution of cigarette smoking was significantly different between cases and controls (P<0.0001), with smokers being overrepresented among the case group. Consequently, all of these variables were further adjusted for multivariate regression analysis. We also found significant differences in rs3841324 between the NPC cases and healthy controls (P = 0.021).

**Table 1:** Frequency Distribution of Demographic and Risk Factors in NPC Cases and Controls.

Variable	Cases (No. = 400)		Controls (No. = 491)		*P*-value*
	No.	(%)	No.	(%)	
**Age (years)**					
<40	63	15.8	105	21.4	0.061
40–60	278	69.5	329	67.0	
>60	59	14.7	57	11.6	
**Sex**					
Male	286	71.5	336	68.4	0.321
Female	114	28.5	155	31.6	
**Smoking Status**					
Ever	158	39.5	126	25.7	<0.0001
Never	242	60.5	365	74.3	
**Drinking Status**					
Ever	116	29.0	123	25.1	0.186
Never	284	71.0	368	74.9	
***CHRNA5*** ** rs3841324**					
Ins/Ins	194	48.5	283	57.6	0.021
Ins/Del	172	43.0	169	34.4	
Del/Del	34	8.5	39	8.0	

We also test the genotype distributions and allele frequencies of *CHRNA5* rs3841324 in the NPC cases and cancer-free controls. The ins and del allele frequencies were in Hardy-Weinberg equilibrium for both cases and controls (χ^2^ = 0.227, P = 0.634 and χ^2^ = 3.619, P = 0.057, respectively). The del allele was more common among the cases (30%) than controls (25.3%), and this difference was statistically significant (χ^2^ = 5.216, P = 0.022), implying that the del allele represented a risk factor for NPC. Similarly, the difference in the *CHRNA5* ins/ins, ins/del, and del/del genotype distributions between the NPC cases and controls were statistically significant (χ^2^ = 7.762, P = 0.021) and the ins/del heterozygote was more common among the NPC cases than among the controls, the difference were statistically significant (χ^2^ = 7.676, P = 0.006). In contrast, although the del/del homozygotes were more common among the cases (8.5%) than controls (8.2%), this difference did not reach statistical significance (χ^2^ = 0.909, P = 0.340). Compared to the ins/ins homozygotes, the ins/del heterozygotes were associated with a 55% elevated risk (adjusted OR = 1.55; 95% CI, 1.17–2.07) for NPC, and the del/del homozygotes also had an increased risk for NPC, although not statistically significant (adjusted OR = 1.34; 95% CI, 0.81–2.23). Furthermore, we found a significant dose-response relationship between the number of del alleles and the risk of NPC (P = 0.011).

To further evaluate the NPC risk associated with the *CHRNA5* polymorphism, we performed stratification analysis by age, gender, cigarette smoking, alcohol consumption, and pathology. Because the del/del variant homozygotes were relatively uncommon, we combined the del/del variant homozygotes with the ins/del variant heterozygotes for this stratification analysis (**Table 2**). Compared with the ins/ins genotypes, the combined variant genotypes (ins/del+del/del) exhibited a >1.5-fold increased risk for NPC (adjusted OR = 1.52; 95% CI, 1.16–2.00). In addition, the increased NPC risk associated with the combined variant genotypes (ins/del+del/del) was evident for ever-smokers (adjusted OR = 2.07; 95% CI, 1.23–3.48), middle-aged subjects (40–60 years of age; adjusted OR = 1.60; 95% CI, 1.14–2.25), never-drinkers (adjusted OR = 1.53; 95% CI, 1.11–2.09), males (adjusted OR = 1.66; 95% CI, 1.19–2.32), or for non-keratinizing carcinoma (adjusted OR = 1.52; 95% CI, 1.15–2.00). We also found a borderline significant interaction between *CHRNA5* rs3841324 genotypes and smoking status (P = 0.080), and a significant interaction between *CHRNA5* rs3841324 genotypes and age (P = 0.002); however, there was no evidence for an interaction between the *CHRNA5* variant genotypes and gender (P = 0.373) or drinking status (P = 0.510) on the risk of NPC. Furthermore, we examined the joint effect of the *CHRNA5* rs3841324 genotype and smoking status on the risk of NPC and found that ever-smokers with the *CHRNA5* rs3841324 ins/del or del/del allele had a >4-fold increased risk for NPC (adjusted OR = 4.35; 95% CI, 2.57–7.38) compared with non-smokers homozygous for the ins allele (**Table 3**).

**Table 2:** Stratification analysis of *CHRNA5* rs3841324 genotype, OR, and 95% CIs by selected variables.

	Cases (No. = 400)				Controls (No. = 491)						Crude OR (95%CI)^a^		Adjusted OR (95%CI)^a^		
	Ins/Ins		Ins/Del + Del/Del			Ins/Ins		Ins/Del + Del/Del		Ins/Ins		Ins/Del + Del/Del	Ins/Ins	Ins/Del + Del/Del	
	No.	(%)	No.	(%)		No.	(%)	No.	(%)						
All	194	48.5	206	51.5		283	57.6	208	42.4	1.00		1.45 (1.11–1.88)	1.00	1.52 (1.16–2.00)	
**Age (years)**															
<40	32	50.8	31	49.2		74	70.5	31	29.5	1.00		2.31 (1.21–4.42)	1.00	1.77 (0.79–3.98)	
40–60	129	46.4	149	53.6		179	54.4	150	45.6	1.00		1.38 (1.00–1.90)	1.00	1.60 (1.14–2.25)	
>60	33	55.9	26	44.1		30	52.6	27	47.4	1.00		0.88 (0.42–1.82)	1.00	0.85 (0.38–1.82)	
**Sex**															
Male	140	49.0	146	51.0		204	60.7	132	39.3	1.00		1.61 (1.17–2.22)	1.00	1.66 (1.19–2.32)	
Female	54	47.4	60	52.6		79	51.0	76	49.0	1.00		1.16 (0.71–1.87)	1.00	1.16 (0.70–1.93)	
**Smoking Status**															
Ever	79	50.0	79	50.0		86	68.3	40	31.7	1.00		2.15 (1.32–3.50)	1.00	2.07 (1.23–3.48)	
Never	115	47.5	127	52.6		197	54.0	168	46.0	1.00		1.30 (0.94–1.79)	1.00	1.33 (0.95–1.85)	
**Drinking Status**															
Ever	56	48.3	60	51.7		66	53.7	57	46.3	1.00		1.24 (0.75–2.06)	1.00	1.67 (0.92–3.03)	
Never	138	48.6	146	51.4		217	59.0	151	41.0	1.00		1.56 (1.11–2.08)	1.00	1.53 (1.11–2.09)	
**Pathology**															
WHO I	7	46.7	8	53.3		283	57.6	208	42.4	1.00		1.56 (0.56–4.36)	1.00	1.65 (0.58–4.73)	
WHO II	187	48.6	198	51.4		283	57.6	208	42.4	1.00		1.44 (1.10–1.88)	1.00	1.52 (1.15–2.00)	

a Adjusted for age, sex, smoking status and alcohol drinking in a logistic regression model.

**Table 3:** Joint effect of *CHRNA5* rs3841324 genotypes and smoking status on risk of NPC.

SmokingStatus	rs3841324genotype	Cases(No. = 400)	Controls(No. = 491)	Crude OR(95%CI)^a^	Adjusted OR(95%CI)^a^
		No. (%)	No. (%)		
Never	Ins/Ins	115 (36.9)	197 (63.1)	1.00	1.00
Never	Ins/Del + Del/Del	127 (43.1)	168 (56.9)	1.30 (0.94–1.79)	1.33 (0.95–1.85)
Ever	Ins/Ins	79 (47.9)	86 (52.1)	1.57 (1.07–2.31)	1.47 (0.95–2.27)
Ever	Ins/Del + Del/Del	79 (66.4)	40 (33.6)	3.38 (2.17–5.28)	4.35 (2.57–7.38)

a adjusted for age, sex, smoking and alcohol accordingly.

## Discussion

CHRNA5, located at the 15q25.1 lung cancer susceptibility locus [5], is an attractive candidate gene for smoking behavior, nicotine dependence, and smoking-related disease, as they have been plausibly linked with carcinogenesis [20], [21]. In this case-control comparison study, we found a significant association between CHRNA5 rs3841324 variant genotypes and NPC risk, and individuals with CHRNA5 rs3841324 combined variant genotypes (ins/del+del/del) had an elevated risk compared to the CHRNA5 ins/ins genotype, especially among ever-smokers. In addition, the combined variant genotypes (ins/del+del/del), in conjunction with cigarettes smoking, increased NPC risk, thus indicating that the CHRNA5 rs3841324 variant not only could be a risk factor and a novel biomarker for prediction of smoking related NPC risk, but also might be involved in the etiology of cigarette smoking-mediated cancer. A possible explanation for this finding is that the 22 bp insertion at the position −71 deletion upstream of the transcription start site change results in an alteration of transcription factor binding sites [22] and DNA- protein interactions [16], and thereby affects the CHRNA5 mRNA level and transcription [15].

It is biologically plausible that genetic variations in the 15q25.1 lung cancer susceptibility locus not only affects risk of smoking-associated cancers directly, but also acts jointly with smoking habits for the etiology of NPC. First, the 15q25.1 locus has been verified to be associated with smoking behavior, persistence, and nicotine dependence [23], thereby influencing the intensity, duration, and cumulative consumption of cigarette exposure, and consequently increases the incidence of smoking-associated diseases, including smoking-associated cancers. In addition, it is through the acetylcholine receptor pathway, which was composed of CHRNA5 and CHRNA3 in the 15q25.1 locus, that nicotine promote cancer cell proliferation, survival, migration, invasion, and tumor etiology and development [3]. Therefore, variants in the 15q25.1 locus have been implicated with nicotine-mediated cancers.

This is the first molecular epidemiologic study to investigate the association between CHRNA5 rs3841324, a susceptible variation for lung cancer, and susceptibility to smoking-associated cancer in addition to lung cancer. The findings of the current study may provide novel information on the etiology of smoking-mediated cancers, verify the role of the 15q25.1 lung cancer susceptibility locus in the risk of smoking-associated cancers, and elucidate the carcinogenic mechanisms by which genetic variants at these regions influence cancer risk. As an oncogenic agent, smoking is estimated to cause approximately 31% and 6% of all cancer deaths in men and women worldwide for people between 30 and 69 years, respectively [2]. These types of cancers include lung cancer [24], NPC [4], oral cavity cancer [25], esophageal cancer [26], colorectal cancer [27], invasive cervical cancer [28], skin cancer [29], prostate cancer [30], kidney cancer [31], breast cancer [32], bladder cancer [33], and pancreatic cancer [34]. Evaluation of the impact of genetic variants on smoking-related disease, especially on smoking-mediated cancers, is of value to public health [20], [35].

Several GWAS have demonstrated that genetic variants in CHRNA5 and CHRNA3, located at the 15q25.1 lung cancer susceptibility locus [20], [21], influence the risk of developing lung cancer in European and US populations [5], [21]. Since then, three studies have examined the associations between the CHRNA5 rs3841324 variant and lung cancer risk, with inconsistent results [6], [16], [36]. For example, Amos *et al.* (6) found that in ever-smokers, homozygous carriers of del variants of rs3841324 exhibited a decreased lung cancer risk in females, but had little effect in males in 624 lung cancer patients and 766 healthy controls from a Caucasian population. A study explored the association between the CHRNA5 rs3841324 polymorphism and lung cancer risk in a Han Chinese population and found no significant association, but revealed that 2 least frequent haplotypes, rs3841324 ins/rs3829787 T and rs3841324 del/rs3829787 C were statistically decrease lung cancer risk [16]. Recently, another study with a Caucasian population of Norwegian origin also showed that rs3841324 statistically influenced the risk of developing lung cancer and a significantly reduced risk of developing non-small-cell lung cancer exists for del allele homozygotes, especially lung tumors harboring a mutated TP53 [36].

Interestingly, in our studies, CHRNA5 rs3841324 combined variant genotypes (ins/del+del/del) had an increased NPC risk compared to ins allele homozygotes, which is inconsistent with the results for lung cancer in which the del allele was a protective factor for a Caucasian population [6], [36]. The discrepancy may result from different tumor sites and ethnicities studied. Previous studies have demonstrated that del allele homozygotes at rs3841324 have a 2.9-fold increase in CHRNA5 mRNA levels in the frontal cortex and that low levels of CHRNA5 mRNA are associated with a lower risk for nicotine dependence and lung cancer [15]. Therefore, del allele homozygotes at rs3841324 are more likely to be at increased risk for nicotine dependence and smoking persistence with increased CHRNA5 mRNA levels in the frontal cortex, and is consequently more likely to be associated with a higher incidence of smoking-mediated cancers. In addition, a study [16] conducted in a Han Chinese population showed no significant association between CHRNA5 rs3841324 variants and lung cancer risk in contrast with the reports conducted in a Caucasian population [6], [36], which suggested the effect of various ethnicities studied. Other studies have demonstrated that variants at the 15q25.1 locus, including rs3841324, may contribute differently to nicotine dependence [37] and cigarette use [38] in European-Americans and African-Americans, which also verifies the effect of diverse ethnicities. Finally, the role of rs3841324 in the etiology of the smoking related cancers has not been confirmed in basic biologic research and rs3841324 might just be a marker in linkage disequilibrium with different causal variants in various smoking related cancers, which also might contribute to the discrepancy in the direction of association. These hypotheses, however, need to be confirmed in future studies.

The current study is the first to report the combined effect of exposure to cigarette smoke and genetic variants at the 15q25.1 lung cancer susceptibility locus on the risk of smoking-mediated cancer. Smokers who had ins/del or del/del genotypes were >4-fold more likely to develop NPC than non-smokers homozygous for the insertion allele, which was much higher than the effect of smoking exposure or genetic variants at the 15q25.1 lung cancer susceptibility locus alone. This findings suggest that genetic variants at the 15q25.1 lung cancer susceptibility locus is an independent risk factor influencing inheritance susceptibility and is also plausible to be a cofactor of cigarettes. Genetic susceptibility and environmental exposure for NPC risk has been widely verified in various populations [39]. However, few case-control comparison studies have investigated the interaction of hereditary predispositions and smoking exposure in the etiology of NPC. Nevertheless, there are three facts which support our findings. First, the biologic mechanism underlying how cigarettes and genes interact at the 15q25.1 lung cancer susceptibility locus is somewhat clear. The fact that tobacco alkaloid and nicotine can bind and activate the nicotinic acetylcholine receptors, and thereby stimulate cellular proliferation and tumor invasion, and inhibit apoptosis [9]–[11], might partly explain the synergism between exposure to cigarette smoking and the 15q25.1 locus on the etiology of NPC. Second, variants at the 15q25.1 lung cancer susceptibility locus could have an effect on smoking persistence [12], smoking behavior, and nicotine dependence [13]. Finally, studies in bladder cancer [40], lung cancer [41], and NPC [4] have demonstrated the combined effect of exposure to cigarette smoking and heredity also partly support our findings.

Another important finding in our study was that the risk of NPC associated with rs3841324 genotypes harboring del alleles was significantly higher for males. The plausible explanation for this observation might be attributed to males being more strongly associated with cigarette smoking. A much higher proportion of males smoke cigarettes and have smoking-associated cancer deaths than females worldwide [2]. In the current study, the increased NPC risk associated with genetic variation at the 15q25.1 locus in ever-drinkers was nearly equal to never-drinkers. We speculate that the discrepancy is partly attributed to the sample sizes in the ever- and never-drinkers because NPC risk was not associated with alcohol consumption [4].

Although conducted in a large, well-characterized case-control comparison, our study had several limitations. First, possible limitations in our hospital-based case-control study design could have introduced some selection bias because subjects were not from the same population. However, all of the subjects were Han Chinese and both areas in which the hospitals were located are low NPC risk regions in China. In addition, we have found that the frequency of the *CHRNA5* genotypes in cases and controls between the two hospitals was not statistically different, and the frequency of the *CHRNA5* genotypes in the healthy control group in the current study was similar to those in previous reports with Han Chinese subjects [16]. Second, the number of observations in some strata of the stratification analysis was relatively small; however, after adjustment for age, gender, cigarette smoking, and alcohol consumption the potential impact from confounding factors on ORs might be minimized. Another possible source of bias in our study might result from the bias of self-reported exposure histories, including histories of cigarette smoking and alcohol consumption. To confirm the role of genetic variants on the 15q25.1 lung cancer susceptibility locus in smoking-associated cancers and whether or not it could be a biomarker for identification of smoking-associated cancers, further studies should be investigated with larger sample sizes in different populations involving other anatomic sites of cancers.

In conclusion, our study demonstrated that genetic variants of the 15q25.1 lung cancer susceptibility locus, rs3841324, are significantly associated with NPC, a smoking-associated cancer other than lung cancer. This observation was especially pertinent to smokers, and could act jointly with cigarette smoking to increase NPC risk, indicating that this genetic variation can serve as a biomarker for the early identification of at-risk populations for smoking-associated cancers. We also noted that the association was most evident in males. This is the first time the role of genetic variants at the 15q25.1 lung cancer susceptibility locus and NPC risk has been examined, which is of value. Further validation of our findings by larger studies and exploration of the underlying molecular mechanisms are warranted.
